# Women cross the ‘Catalina Channel’ faster than men

**DOI:** 10.1186/s40064-015-1086-4

**Published:** 2015-07-08

**Authors:** Beat Knechtle, Thomas Rosemann, Christoph Alexander Rüst

**Affiliations:** Gesundheitszentrum St. Gallen, Vadianstrasse 26, 9001 St. Gallen, Switzerland; Institute of Primary Care, University of Zurich, Zurich, Switzerland

**Keywords:** Ultra-endurance, Women, Men, Sex difference

## Abstract

Open-water ultra-distance swimming has a long history where the ‘English Channel’ (~33 km) was crossed in 1875 for the first time. Nowadays, the three most challenging open-water swims worldwide are the 21-miles (34 km) ‘English Channel Swim’, the 20.1-miles (32.2 km) ‘Catalina Channel Swim’ and the 28.5-miles (45.9 km) ‘Manhattan Island Marathon Swim’, also called the ‘Triple Crown of Open Water Swimming’. Recent studies showed that women were able to achieve men’s performance in the ‘English Channel Swim’ or to even outperform men in the ‘Manhattan Island Marathon Swim’. However, the analysis of the ‘Catalina Channel Swim’ as part of the ‘Triple Crown of Open Water Swimming’ is missing. We investigated performance and sex difference in performance for successful women and men crossing the ‘Catalina Channel’ between 1927 and 2014. The fastest woman ever was ~22 min faster than the fastest man ever. Although the three fastest women ever were ~20 min faster than the three fastest men ever, the difference reached not statistical significance (*p* > 0.05). Similarly for the ten fastest ever, the ~1 min difference for women was not significant (*p* > 0.05). However, when the swimming times of the annual fastest women (*n* = 39) and the annual fastest men (*n* = 50) competing between 1927 and 2014 were compared, women (651 ± 173 min) were 52.9 min (16 ± 12%) faster than men (704 ± 279 min) (*p* < 0.0001). Across years, swimming times decreased non-linearly in the annual fastest men (polynomial 2nd degree) and women (polynomial 3rd degree) whereas the sex difference decreased linearly from 52.4% (1927) to 7.1% (2014). In summary, the annual fastest women crossed the ‘Catalina Channel’ faster than the annual fastest men. The non-linear decrease in swimming times suggests that female and male swimmers have reached a limit in this event. However, the linear decrease in the sex difference may indicate that women continuously narrow the gap to men.

## Background

Open-water ultra-distance swimming has a very long tradition (Eichenberger et al. 2012a; Fischer et al. 2013; Knechtle et al. 2014a, b) and there seem nearly no limits in the length of the covered distances (De Ioannon et al. 2015; Drygas et al. 2014). Long-distance open-water swimming started in 1875 when Captain Matthew Webb was the first man to cross the ‘English Channel’ in 21:45 h:min (www.history.com/this-day-in-history/captain-webb-swims-english-channel). Around 50 years later in 1926, the New Yorker Gertrud Ederle was the first woman to cross the ‘English Channel’ in 14:32 h:min and to become overnight a hero in the United States (www.history.com/this-day-in-history/gertrude-ederle-becomes-first-woman-to-swim-english-channel).

The US-American William Wrigley was particularly interested in the success of Gertrud Ederle in the ‘English Channel’ and launched the first open-water ultra-distance swimming event in the United States of America. He created the ‘Wrigley Ocean Marathon Swim’ in California from Avalon on Santa Catalina Island to Point Vicente, a landmark on the Californian coast (http://swimcatalina.com/images/stories/history/catalinachannelhistory.pdf). The straight line across the San Pedro Channel was 22 miles (35 km), just one mile longer than the official ‘English Channel’.

On January 15, 1927, a total of 102 swimmers (87 men and 15 women) started for the first ‘Wrigley Ocean Marathon Swim’ where only one man finished. George Young won the first edition across the ‘Catalina Channel’ in 15:44:30 h:min:s. Within 3 months of the first ‘Wrigley Ocean Marathon Swim’, four swimmers successfully tackled the ‘Catalina Channel’ (http://swimcatalina.com/images/stories/history/catalinachannelhistory_wrigley_marathon_swim.pdf). Since the first edition in 1927 until the end of 2014, a total of 370 swimmers successfully crossed the ‘Catalina Channel’ (http://swimcatalina.com/index.php/successful-swims/successes).

The ‘Catalina Channel’ can be crossed like the ‘English Channel’ in two directions, from Catalina to the mainland (CM) or from the mainland to Catalina (MC). For both directions, the fastest woman ever was faster than the fastest man ever. For CM, Grace Van Der Byl (7:27:25 h:min:s on October 5, 2012) was ~38 min faster than Todd Robinson (8:05:44 h:min:s on August 25, 2009). For MC, Penny Lee Dean (7:15:55 h:min:s on September 1, 1976) was ~22 min faster than Pete Huisveld (7:37:31 h:min:s on August 20, 1992) (http://swimcatalina.com/index.php/successful-swims/individuals).

Apart from the ‘Manhattan Island Marathon Swim’, the ‘Catalina Channel Swim’ is the only major channel crossing on the American continent and is comparable to the ‘English Channel Swim’ in both distance and difficulty. Furthermore, the ‘Catalina Channel Swim’ is part of the ‘Triple Crown of Open Water Swimming’. The ‘Triple Crown of Open Water Swimming’ (www.triplecrownofopenwaterswimming.com) includes three of the most well-known marathon swims. These are the 21-miles (34 km) ‘English Channel Swim’ in England, the 20.1-miles (32.2 km) ‘Catalina Channel Swim’ in the United States and the 28.5-miles (45.9 km) ‘Manhattan Island Marathon Swim’ also held in the United States. The fastest completion time for the ‘Triple Crown of Open Water Swimming’ (i.e. all three events together) by the end of 2014 was set in 2008 by the female US-American ultra-distance swimmer Rendy Lynn Opdycke (www.teamusa.org/Athletes/OP/RendyLynn-Opdycke.aspx). She completed all the three events in 70:50 h:min within 36 days during July and August 2008.

Recent studies investigated participation and performance trends for the ‘English Channel Swim’ (Eichenberger et al. 2012a; Fischer et al. 2013) and the ‘Manhattan Island Marathon Swim’ (Knechtle et al. 2014a). The analysis of the ‘Catalina Channel Swim’ is, however, still missing. Women seemed to achieve similar or even better performances in two of the three events in the ‘Triple Crown of Open Water Swimming’. The fastest women were faster than the fastest men in the ‘Manhattan Island Marathon Swim’ (Knechtle et al. 2014a) and the best annual performances did not differ between the sexes in the ‘English Channel Swim’ (Eichenberger et al. 2012a). Based upon these findings for ‘Manhattan Island Marathon Swim’ (Knechtle et al. 2014a) and the ‘English Channel Swim’ (Eichenberger et al. 2012a) and the fact that the fastest women ever were faster than the fastest men in ‘Catalina Channel Swim’ (http://swimcatalina.com/index.php/successful-swims/individuals), we hypothesized that the analysis of all successful swims in the ‘Catalina Channel Swim’ would show similar results as it has been reported for the ‘Manhattan Island Marathon Swim’ (Knechtle et al. 2014a) and the ‘English Channel Swim’ (Eichenberger et al. 2012a).

## Methods

### The Catalina Channel Swim

The ‘Catalina Channel’ is a little more than 20 miles (32.2 km) at its shortest distance and can be crossed from Catalina to the mainland (CM) or from the mainland to Catalina (MC). Like the ‘English Channel Swim’, the ‘Catalina Channel Swim’ is open to anyone and can be tackled at any time of the year (http://swimcatalina.com/). The ‘Catalina Channel Swim’ is a non-wetsuit event like all other long-distance swims. The athletes attempt the swim usually between late spring and the end of the summer when the water temperature varies from 15 to 21°C (http://swimcatalina.com/).

Each interested swimmer and his support crew must follow both the general and the additional rules set by the official ‘CATALINA CHANNEL SWIMMING FEDERATION’ (CCSF) (http://swimcatalina.com/images/stories/forms/ccsf_rules_2015.pdf). Every swimmer must be supported by a guide kayak to provide food, drink and navigation. Generally, the swimmers start at midnight to avoid the blustery afternoon winds. Swimming in the complete dark needs practice to learn how to navigate with glow sticks as the only guide (http://swimcatalina.com/index.php/information/challenges-of-catalina).

Water temperatures in the ‘Catalina Channel’ may fluctuate from 1 day to the next (http://ca.usharbors.com/harbor-guide/avalon-santa-catalina-island/weather). Surface water temperatures may change throughout the day and are colder in the morning and warmer under the afternoon sun (http://marathonswimmers.org/blog/2012/03/catalina-water-temp/). Generally, the water in the ‘Catalina Channel’ is warmer near the island. Often, the temperature drops several degrees (~5°C) in the three miles nearest the California mainland. Currents in the ‘Catalina Channel’ are unpredictable and can be swift. The surface in the ‘Catalina Channel’ can be influenced by ocean currents impacting the speed of the swimmers, particularly the slower ones’ (http://www.sccoos.org/data/hfrnet/). The Pacific Ocean rolls through the ‘Catalina Channel’ and swells of 1.5–2.5 m are common. The rolling motion may lead to seasickness (http://swimcatalina.com/index.php/information/challenges-of-catalina).

### Data sampling and data analysis

The data set for this study was obtained from the official race website from the ‘CATALINA CHANNEL SWIMMING FEDERATION’ (http://swimcatalina.com/). Swim times from all 370 successful female and male swimmers crossing the ‘Catalina Channel’ between 1927 and 2014 were considered.

### Statistical analysis

Data were tested for normal distribution and homogeneity of variances prior to statistical analyses. Normal distribution was tested using D’Agostino and Pearson omnibus normality test and homogeneity of variances was tested using Levene’s test. Single level regression analyses were used to investigate changes in performance and sex difference in performance of the finishers. Since the change in sex difference in endurance is assumed to be non-linear (Reinboud 2004), we also calculated the non-linear regression model that fits the data best. We compared for each variable (i.e. swim times, sex difference in swim times) the best-fit non-linear regression model to the linear model using Akaike’s Information Criteria (AIC) in order to show which model would be the most appropriate to explain the trend of the data. To find differences between two groups, e.g. between men and women, a Student’s *t* test was used in case of normal distributed data (with Welch’s correction in case of unequal variances) and a Mann–Whitney test was used in case of not normal distributed data. Statistical analyses were performed using IBM SPSS Statistics (Version 22, IBM SPSS, Chicago, IL, USA), CurveExpert Professional (Version 2.0.3, Hyams D.G.) and GraphPad Prism (Version 6.01, GraphPad Software, La Jolla, CA, USA). Significance was accepted at *p* < 0.05 (two-sided for *t* tests). Data in the text and figures are given as mean ± standard deviation (SD).

## Results

Between 1927 and 2014, a total of 370 swimmers with 135 women (36.5%) and 235 men (63.6%) crossed the ‘Catalina Channel’. More swimmers were crossing from Catalina to mainland (CM, 338, 91.3%) than from mainland to Catalina (MC, 32, 8.7%). On average, ~7 swimmers were successful per calendar year with ~3 to 4 women and ~4 to 5 men with a men-to-women ratio of ~2 (Table 1). The number of female (r^2^ = 0.28, *p* = 0.0005) and male (r^2^ = 0.33, *p* < 0.0001) swimmers increased across calendar years. The men-to-women ratio, however, remained unchanged (r^2^ = 0.01, *p* = 0.55). The largest increase of successful swimmers was in the last 10 years. Most of the successful finishers originated from the United States (76.2%), followed by swimmers from Great Britain (4.3%), Australia (3.5%), Mexico (2.9%) and Canada (2.1%) (Table 2).

**Table 1:** The number of successful female and male finishes with the men-to-women ratio

Year	All	Women	Men	Men-to-women ratio
1927	5	1	4	4
1936	1		1	
1946	1		1	
1952	3	1	2	2
1953	2		2	
1954	1		1	
1955	3	2	1	0.5
1956	4	1	3	3
1957	3		3	
1958	2	2		
1959	3	2	1	0.5
1963	1		1	
1971	4	2	2	1
1972	3	1	2	2
1974	3	1	2	2
1976	4	2	2	1
1977	7	5	2	0.4
1978	2		2	
1979	2	1	1	1
1980	3	1	2	2
1981	2	1	1	1
1982	4	1	3	3
1983	3	1	2	2
1984	9	4	5	1.25
1985	3	1	2	2
1986	4		4	
1987	2	1	1	1
1989	2	1	1	1
1990	2		2	
1991	4		4	
1992	4	1	3	3
1993	5		5	
1994	4	2	2	1
1995	4	2	2	1
1997	3	2	1	0.5
1998	4	1	3	3
1999	3		3	
2000	4	1	3	3
2001	5	3	2	0.67
2002	2	2		
2003	5		5	
2004	4	1	3	3
2005	12	1	11	11
2006	13	6	7	1.167
2007	8	5	3	0.6
2008	25	14	11	0.78
2009	16	2	14	7
2010	29	11	18	1.63
2011	26	9	17	1.88
2012	24	9	15	1.66
2013	39	14	25	1.78
2014	39	17	22	1.29

**Table 2:** The nationalities of the successful swimmers, sorted by the number of successful crossings

Country	Number of successful athletes
United States of America	282
Great Britain	16
Australia	13
Mexico	11
Canada	8
Spain	5
Ireland	5
Iran	4
India	4
New Zealand	4
Cuba	3
Italy	2
Egypt	2
South Africa	2
Scotland	2
Jersey Island	1
Hungary	1
Guatemala	1
Greece	1
Sweden	1
France	1
Brazil	1

The fastest woman ever was ~22 min faster than the fastest man ever (Table 3). Although the three fastest women ever were ~20 min faster than the three fastest men ever, the difference reached not statistical significance (*p* > 0.05). Similarly, for the ten fastest ever, the ~1 min difference for women was not significant (*p* > 0.05). When all female swim times were compared to all male swim times, women were ~31 min faster, but the difference was again not significant (*p* > 0.05).

**Table 3:** Swim times for the fastest ever, the three fastest ever, the ten fastest ever and all women and men between 1927 and 2014

	Women (min)	Men (min)	Difference (min)	*p*
Fastest ever	435	457	22	
Three fastest ever	448.8 ± 7.8	468.1 ± 8.8	19.3 ± 11.8	0.885
Ten fastest ever	489.8 ± 9.6	490.8 ± 6.0	0.9 ± 11.4	0.1781
All	707.6 ± 166.2	738.8 ± 215.7	31.2 ± 49.5	0.66

In 39 calendar years, at least one woman crossed the ‘Catalina Channel’, and in 50 calendar years at least one man. When the swimming times of the annual fastest women (*n* = 39) and the annual fastest men (*n* = 50) competing between 1927 and 2014 were compared, women (651 ± 173 min) were 52.9 min (16 ± 12%) (*t* = 4.31, *p* < 0.0001) faster than men (704 ± 279 min) (Table 4). When we consider only those calendar years where both one women and one man were crossing ‘Catalina Channel’ (*n* = 37), women (652 ± 177.85 min) were not faster (*p* = 0.9634) than men (654.30 ± 188.97 min).

**Table 4:** Comparison of swim times between the annual fastest women and men competing between 1927 and 2014 and the sex difference in performance

Year	Women (min)	Men (min)	Women faster than men (%)	Men faster than women (%)
1927	1,242	815		52.4
1936		2,030		
1946		1,340		
1952	827.5	1,100	24.8	
1953		998		
1954		805.7		
1955	1,024	1,134	9.78	
1956	940	550.5		70.7
1957		938		
1958	649.7			
1959	667.5	1,255	46.8	
1963		765		
1971	754	754		
1972	738	530		39.2
1974	528	620	14.8	
1976	435.9	529.8	17.7	
1977	513	511.3		0.3
1978		461.2		
1979	603.5	734	17.8	
1980	662.2	722	8.3	
1981	677.6	688	1.5	
1982	719.7	548		31.3
1983	938.6	507.4		84.9
1984	568.5	534		6.4
1985	566	808.8	30	
1986		673.4		
1987	615	634.3	3.1	
1989	893.4	795.7		12.3
1990		696.7		
1991		608		
1992	659.4	457.5		44.1
1993		494.8		
1994	463.1	661.6	30.1	
1995	767.1	646.8		18.6
1997	632.7	804.9	21.4	
1998	523	851	38.5	
1999		608.6		
2000	765.5	512.8		49.3
2001	513.3	568.5	9.7	
2002	623.2			
2003		611.2		
2004	576.6	619.4	6.9	
2005	536.2	536.2		
2006	500.8	584.3	14.3	
2007	566.8	531.9		6.6
2008	508.4	554.4	8.3	
2009	658.5	485.7		35.6
2010	516.5	487.1		6.1
2011	586.5	498.5		17.7
2012	447.4	491.2	8.9	
2013	484.2	573.8	15.6	
2014	531.3	571.6	7.1	
	651.91 ± 173.17	704.79 ± 279.42	16.7 ± 12.0	31.7 ± 25.3

In 20 calendar years, the annual fastest woman was faster than the annual fastest man. In 15 calendar years, the annual fastest man was faster than the annual fastest women. In the 20 calendar years with the faster women, the annual fastest women (589.40 ± 139.67 min) were 135.93 min (16.79 ± 12.01%) faster than the annual fastest men (725.33 ± 213.55 min) (*p* < 0.0001). In the 15 calendar years with the faster men, the annual fastest men (560.81 ± 107.96) were 177.42 min (31.70 ± 25.31%) faster than the annual fastest women (738.23 ± 198.98) (*p* < 0.0001).

**Figure 1:**
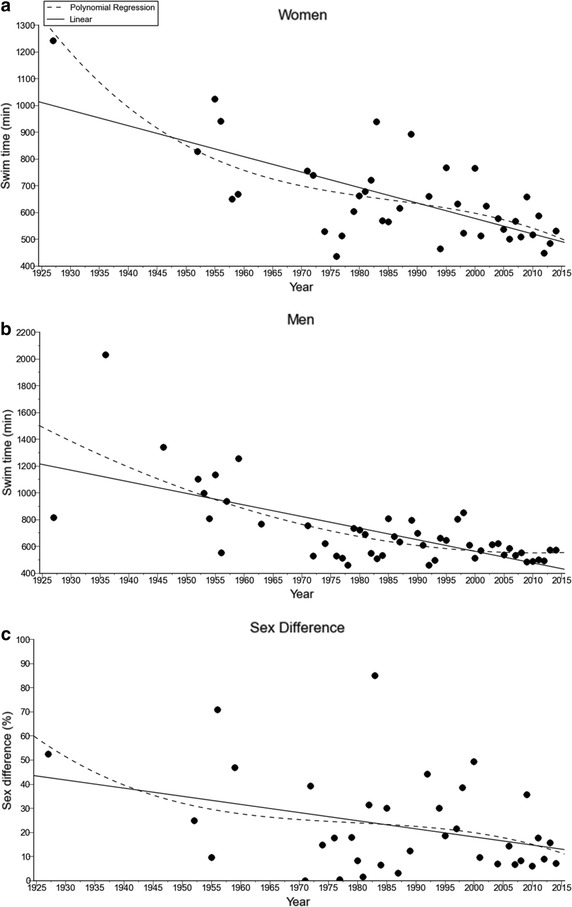
Swimming time (min) for the annual fastest women (**a**) and men (**b**) with the sex difference in swimming (**c**) for the annual fastest.

Across years, both women and men became faster. Swimming times decreased non-linearly in men (i.e. polynomial 2nd degree) and in women (i.e. polynomial 3rd degree) across calendar years whereas the sex difference decreased linearly (*r* = 0.36) from 52.4% in 1927 to 7.1% in 2014 (Figure 1). Table 5 presents the results of the comparison between the linear and the non-linear regressions.

**Table 5:** The comparison between the linear and the non-linear model

	Sum of squares	DOF	AIC	Delta	Probability	Likelihood
Women						
Polynomial	504,044	35	375.893	2.80745	0.197226	80.27%
Linear	609,125	37	378.701			
Men						
Polynomial	1.8982e+06	47	531.475	3.19464	0.168357	83.16%
Linear	2.11327e+06	48	534.67			
Sex difference						
Polynomial	12,240.6	32	216.594	3.81782	0.129104	87.08%
Linear	12,520.7	34	212.776			

## Discussion

This study investigated the performance and sex difference in performance for successful swimmers crossing the ‘Catalina Channel’ since 1927. The most important findings were, first, the annual fastest women were faster than the annual fastest men, second, the sex difference in swimming times decreased linearly across years although swimming times decreased non-linearly.

### Women were faster than men

The most important finding was that the fastest women ever were faster than the fastest men ever. The differences were non-significantly when the three and the ten fastest women and men ever were compared. The difference became, however, highly significant when the swimming times of the annual fastest women and men were compared.

We need to consider more closely the investigated samples for the interpretation of these findings. The selection of the swimmers and the kind of the analysis seem to have a considerable influence the outcome of the results. The annual fastest women were ~16 min faster than the annual fastest men, leading to a sex difference in performance of ~2.3%. For this result, we considered all calendar years between 1927 and 2014. However, not in all years at least one woman and one man were crossing the ‘Catalina Channel’. When we consider only those calendar years where at least one woman and one man crossed the ‘Catalina Channel’, mean swim times were 651.9 ± 173.1 min and 653.1 ± 186.5 min for women and men, respectively, which is not statistically different (*p* > 0.05). This result is confirming recent findings for the ‘English Channel Swim’ (Eichenberger et al. 2012a; Fischer et al. 2013).

The comparison of the performances of the best competitors ever leads to different findings in contrast to comparing sex differences over time between the annual fastest. For example, for the ‘English Channel Swim’, Eichenberger et al. (2012a) considered the mean swimming speed of the annual fastest where the sex difference in swimming speed was not different between the annual fastest women and men. Similarly, Fischer et al. (2013) considered the mean swim times of all successful female and male swimmers where no difference in swim times was found. However, when the fastest ever, the three fastest ever and the ten fastest ever in the ‘English Channel Swim’ were considered, men were always faster than women (Fischer et al. 2013). When the annual three fastest women and men in the ‘English Channel Swim’ were compared, men were ~12.5% faster during the 1975–2011 period (Fischer et al. 2013). Different findings were reported, however, for the ‘Manhattan Island Marathon Swim’, where the best women were ~14% and ~12% faster considering the three and the ten fastest ever (Knechtle et al. 2014a). Additionally, for the annual three fastest women and men competing during the 1983–2013 period, women were ~4.5% faster than men (Knechtle et al. 2014a).

In the ‘Catalina Channel Swim’, the fastest woman ever was faster than the fastest man ever, similar to the ‘Manhattan Island Marathon Swim’ (Knechtle et al. 2014a). In the ‘English Channel Swim’, however, the fastest man was faster than the fastest woman (Fischer et al. 2013). These disparate findings for the fastest solo swimmers are difficult to explain. Both the ‘English Channel Swim’ and the ‘Catalina Channel Swim’ are held as solo swims with no possibility for drafting. Furthermore, the distances in both events are nearly identical. Also water temperatures seem very similar during the summer months in both the ‘English Channel’ (www.weatheronline.co.uk/marine/weather?LEVEL=77&MENU=0&MEER=kana) and in the ‘Catalina Channel’ (www.ndbc.noaa.gov/station_page.php?station=46025). It was assumed that the faster performances in women in the ‘Manhattan Island Marathon Swim’ were most likely due to the low water temperatures and the higher body fat in female swimmers (Knechtle et al. 2014a). However, when the water temperatures are very similar in all events in the ‘Triple Crown of Open Water Swimming’, the longer distance in the ‘Manhattan Island Marathon Swim’ is the most likely reason that women were faster than men in that specific event.

Generally, men are faster than women when running was investigated for different distances and for the best performances (Coast et al. 2004; Sparling et al. 1998). The sex difference in running was ~11 to 12% independent on the distance (Coast et al. 2004; Sparling et al. 1998). Similarly, the sex difference in performance was ~11 to 12% when different sports disciplines were investigated (Thibault et al. 2010). However, ultra-endurance performances were not considered in these studies where an ultra-endurance performance is defined as any continuous performance of 6 h or longer in duration (Zaryski and Smith 2005). In ultra-endurance events, men are generally faster than women in cycling (Rüst et al. 2013), running (Zingg et al. 2014a), and triathlon (Knechtle et al. 2014c), but not in swimming (Knechtle et al. 2014a). The sex differences in ultra-distance swimming are generally lower than the 11–12% sex difference for shorter distances (Eichenberger et al. 2012b, 2013; Vogt et al. 2013; Zingg et al. 2014c) and in some instances the performance of female and male long-distance swimmers is equal (Eichenberger et al. 2012a, b) depending upon the kind of data analysis. The most likely reason for the lower or even inexistent sex difference in ultra-distance swimming performance might be explained by anthropometric characteristics such as body fat. Female ultra-distance swimmers have more body fat than male ultra- swimmers (Knechtle et al. 2010) and anthropometric characteristics are related to swimming performance in women, but not in men (Siders et al. 1993). Swimmers with higher body fat stay longer in cold water compared to swimmers with lower body fat (Keatinge et al. 2001).

### The aspect of drafting

From the three events of the ‘Triple Crown of Open Water Swimming’, the ‘English Channel Swim’ and the ‘Catalina Channel Swim’ are held as solo swims where athletes are not able to draft. Drafting behind a leading swimmer reduces drag leading to a reduction of oxygen uptake, heart rate, blood lactate, rating of perceived exertion, and stroke rate and an increase in stroke length (Chatard and Wilson 2003). These adaptations might reduce fatigue for the swimmer behind the leading swimmer and enhance performance in the final stage of an open-water ultra-distance swimming event when held as a race (Rüst et al. 2014a).

However, women were faster than men in the ‘Catalina Channel Swim’ as a non-drafting event and in the ‘Manhattan Island Marathon Swim’ which is held as a draft-legal race. Nonetheless, women were able to swim faster than men. In the ‘Manhattan Island Marathon Swim’, which is held as an annual race, women are able to draft behind men (Knechtle et al. 2014a). Most probably, the swim speed is relatively low during these ultra-swimming races and the benefit to save energy while drafting becomes irrelevant.

### Linear decrease in sex difference across years

Although the decrease in swimming times across years was non-linear, the change in sex difference was linear. A linear change in sex difference may assume that this trend continues in the next years. In other terms, women will narrow the gap to men and due to the linear change women might outrun men in the future. A decrease in sex difference has also been reported for other ultra-distance swimming race such as the ‘La Traversée Internationale du Lac St-Jean’ (Rüst et al. 2014a) and the ‘Maratona del Golfo Capri-Napoli’ (Rüst et al. 2014b). This finding for open-water ultra-distance swimming is different compared to other sports disciplines where the gap between women and men extends to the opposite where women became slower compared to men. For example, in very long distances in ultra-marathon running (Zingg et al. 2014a) and ultra-triathlon (Knechtle et al. 2014c), the sex difference in performance increased across years assuming that women will become slower compared to men.

The sex difference in athletic performance might also be influenced by differences in female and male participation (Capranica et al. 2013). For both women and men, the number of successful finishes increased in the ‘Catalina Channel Swim’ in the last ~10 years. However, the men-to-women ratio remained unchanged. In the ‘English Channel Swim’, the increase in participants was exponential in the last ~40 years (Knechtle et al. 2014b). However, the sex difference remained unchanged for the annual three fastest in this time period. The decrease in sex difference in the ‘Catalina Channel Swim’ in contrast to the unchanged sex difference in the ‘English Channel Swim’ might be due to the different time periods (i.e. ~40 years from 1975 to 2011 compared to ~90 years from 1927 to 2014) and the different samples (i.e. annual fastest compared to annual three fastest).

### Most of the competitors originated from the United States

In this event, most of the successful competitors originated from the United States of America. This finding confirms recent reports that participation was highest for local athletes. For the ‘English Channel Swim’, most of the successful crossings were achieved by British women and men (Knechtle et al. 2014b). However, US-American women and men achieved the fastest times in the ‘English Channel Swim’ (Knechtle et al. 2014b). Generally, US-American swimmers are among the best swimmers world-wide. Regarding pool-swimming, the best swimmers originate from the United States of America and Australia when competing at the Olympic Games (Trewin et al. 2004). However, the participation trend regarding the nationality of the athletes seems different in ultra-distance swimming. In the FINA World Cup races held worldwide, athletes from Italy, Spain Russia, France and Germany mainly competed in the period 2000–2012 (Zingg et al. 2014b, c). In the FINA 10 km open-water races held between 2008 and 2012, most of the competitors were from Brazil and Germany (Vogt et al. 2013). In the ‘Maratona del Golfo Capri-Napoli’ held in Italy, most of the competitors were from Egypt, Italy, Argentina and Syria in the 1954–2013 period (Rüst et al. 2014b). Most probably, the best open-water ultra-distance swimmers travel to these races independent of the venue of the event.

## Conclusions

In summary, the annual fastest women crossed the ‘Catalina Channel’ faster than the annual fastest men. The non-linear decrease in swimming times for both women and men suggests that performance has reached a limit. The linear decrease in sex difference in performance suggests that women will be able to continuously reduce the gap to men.
